# Protection of lithium anodes by fibrous silica nanospheres†

**DOI:** 10.1039/c9ra09481d

**Published:** 2020-01-20

**Authors:** Jinxin Fan, Yu Luo, Keliang Jiang, Cheng Wang

**Affiliations:** Institute for New Energy Materials & Low-Carbon Technologies, School of Material Science and Engineering, Tianjin University of Technology Tianjin 300384 China cwang@tjut.edu.cn; Weifang Institute for Product Quality Inspection Weifang Shandong 261000 China

## Abstract

Lithium metal (anode) has attracted significant attention for use in lithium-metal batteries due to its high energy density, but its practical application is still hindered by the dendrite growth during the battery charging process. Here, fibrous silica nanospheres were prepared *via* a direct hydrothermal reaction and coated on a separator to form a composite electrode with lithium sheets. Upon using this composite electrode in a symmetrical cell, the charge and discharge curves became more stable and the overpotential was alleviated compared with that of the bare lithium metal electrode. Meanwhile, the coulombic efficiency obtained from the Li‖Cu cell remained above 95.9% after 200 cycles at 0.5 mA h cm^−2^. The validity of using this composite electrode in the Li‖LFP (LiFePO_4_, lithium iron phosphate) cells was also evaluated. The results show that the composite electrode can help restrict the growth of lithium dendrites and the accumulation of dead lithium.

## Abstract 2

A fibrous silica nanosphere coating was designed and prepared to protect lithium-metal anodes.

## Graphical abstract

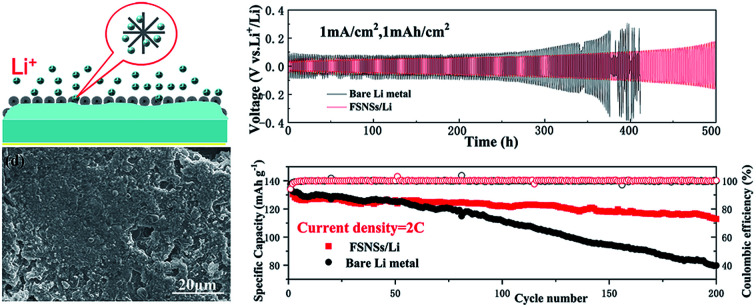

## 1. Introduction

With the rapid development of portable electronic devices, electric vehicles and renewable energy, high-energy-density batteries are highly demanded.^1–7^ Currently, the capacity of lithium-ion batteries (LIBs) with the traditional graphite anode has reached its limit and thus, it is highly necessary to search for high-capacity anode materials. Lithium metal is the best choice for the anode in a lithium-metal battery (LMB) because of its considerably high theoretical specific capacity (3860 mA h g^−1^) and the lowest electrochemical reduction potential (−3.04 V *vs.* standard hydrogen electrode (SHE)).^1,3,8–10^ Nevertheless, during the cycling process, due to the uneven distribution of lithium ions on bare lithium metal (Li metal) electrodes, a local high electric field and concentrated lithium ions can accelerate the nucleation and growth of lithium dendrites.^11–13^ Once lithium dendrites grow to a certain extent, they may pierce the separator and cause safety issues. In addition, the volume change in the lithium anode in the plating/stripping process makes the SEI layer unstable, resulting in the formation of more lithium dendrites, rapid consumption of the electrolyte, and reduction in coulombic efficiency.^14^

In order to solve these problems, scientists have developed the following strategies to stabilize the lithium anode: (1) employing solid electrolytes and modifying liquid electrolytes to improve the stability of the SEI film^4,5,15–19^ or preparing artificial SEI films *via* physical, chemical or electrochemical methods.^6,20,21^ (2) Constructing lithophilic matrices to redistribute lithium ions on the anode surface *via* chemical binding interactions^22–26^ or conductive matrices to effectively reduce the formation of dead Li.^27–31^ (3) Preparing artificial coatings consisting of inorganic (Al_2_O_3_ protective layers^32–37^ and SiO_2_ nanosphere/nanosheet coating layers on the separator^38–41^) or organic (thin Nafion layers^42^) materials. In the case of inorganic SiO_2_ layers, despite their intrinsic insulating nature, they possess numerous polar groups such as O–H and Si–O^22^ that can provide sites for the adsorption or/and deposition of lithium ions, which can facilitate the uniform distribution of lithium ions and avoid their accumulation on preformed tips. Furthermore, silica can electrochemically react with Li through solid-state conversion. This will consume freshly formed Li dendrites and hinder their further growth.^22,40^

**Scheme 1:**
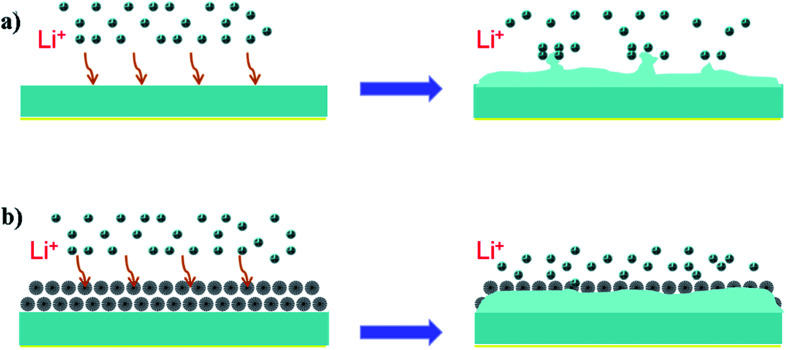
Schematic illustration of Li plating. (a) Li dendrites formed on bare Li-metal electrode and (b) uniform Li deposition on FSNS/Li composite electrode.

In this work, we prepared a simple fibrous silica nanosphere/lithium metal (FSNS/Li) composite electrode for improving the cycling stability of lithium batteries. Fibrous silica nanospheres (FSNSs) were coated on a separator and contacted with lithium sheets *via* mechanical pressing for the fabrication of the FSNS/Li composite electrode. Compared with solid nanometer silicon coatings, in which solid silicon nanospheres were coated on separators to help suppress the formation of Li dendrites,^40,41^ our FSNS/Li composite electrode has at least four advantages (Scheme 1): (1) the thickness of the FSNS coating is about 5 μm, and it is thinner than previously reported coatings (∼20 μm).^40,41^ (2) Fibrous silica nanospheres have a larger specific surface area and can enable more functional groups to participate in the reaction to guide the uniform deposition of lithium ions. (3) The porous structure can provide more space for the deposition of free lithium ions, reducing the deposition of dead lithium on the electrode surface. (4) The pores of the spheres and the gaps among the nanospheres can alleviate the volumetric expansion during the deposition of Li.

## 2. Experimental section

### 2.1 Materials

The main materials used to prepare FSNSs are as follows: tetraethyl orthosilicate (TEOS) was purchased from Fuchen (Tianjin) Chemical Reagents Co., Ltd. Pentanol cetylpyridinium bromide and *N*-methyl-2-pyrrolidone (NMP) were obtained from Shanghai Macklin Bio-Chem Technology Co., Ltd. Cyclohexane, urea and diethyl carbonate (DEC) were purchased from Sinopharm Chemical Reagent Co., Ltd. Copper (Cu) foils and aluminum (Al) foils were purchased from Shenzhen Kejing Star Technology Co., Ltd. Lithium iron phosphate (LiFePO_4_, LFP) powder was purchased from Tianjin B&M Science and Technology Joint-Stock Co., Ltd. All commercial electrolytes used in this experiment were purchased from Suzhou Duoduo Chemical Technology Co., Ltd.

### 2.2 Preparation of porous silica sphere coating

FSNSs were synthesized *via* the conventional hydrothermal reaction. The detailed synthesis procedure is reported in ref. 43 and 44. In a typical process, 2.5 g TEOS was dissolved in a mixed solution of 30 mL cyclohexane and 1.5 mL pentanol. Then, 1.0 g cetylpyridinium bromide, 0.6 g urea and 30 mL ultrapure water were added to this solution. The mixture was stirred for 30 min and then transferred to a 100 mL Teflon-lined stainless steel autoclave. The autoclave was sealed and heated in an oven at 120 °C for 4 h. After hydrothermal treatment, the autoclave was allowed to cool to ambient temperature naturally. The precipitate obtained was washed with ultrapure water and absolute ethanol three times and finally calcined at 550 °C for 6 h in air to yield the target porous silica spheres.

The FSNS coating was prepared by first blending 0.06 g porous silica spheres and 0.01 g PVDF dispersed in 0.5 mL NMP to form a slurry. Then, the slurry was pasted onto a polypropylene (PP) separator and dried in a vacuum oven at 45 °C for 16 h. After drying, the coated diaphragm was cut into a circle with a diameter of 16 mm.

### 2.3 Electrochemical measurements

All cells were assembled in an argon-filled glove box with less than 0.1 ppm O_2_ and 0.1 ppm H_2_O. The Li‖Li, Li‖Cu and Li‖LiFePO_4_ cells were assembled in CR2032 coin cells. The cells were tested using a LAND electrochemical testing system (Wuhan LAND Electronics Co., Ltd.) at room temperature.

For the galvanostatic cycling test in the symmetrical cells, the capacities of the cells were controlled at 1.0 mA h cm^−2^ and the cells were cycled at the current densities of 0.5, 1.0 and 2.0 mA cm^−2^. The cells were assembled using 1.0 M LiPF_6_ (lithium hexafluorophosphate) in a mixture of EC (ethylene carbonate)/DEC (dimethyl carbonate) (1 : 1 by volume) as the electrolyte and polypropylene (PP) coated with porous silica spheres as the separator.

The Li‖Cu cell was fabricated using a piece of Cu foil as the working electrode for Li metal plating. One M LiTFSI (lithium bis(trifluoromethanesulfonyl)imide) in DOL (1,3-dioxolane)/DME (1,2-dimethoxyethane) (1 : 1 by volume) with 1.0 wt% LiNO_3_ was added as the electrolyte. The cells were first cycled between 0 V and 0.5 V at 0.05 mA cm^−2^ for five cycles to stabilize the SEI. Subsequent cycling tests were carried out by depositing 0.5 or 1.0 mA h cm^−2^ of Li onto the Cu current collector and stripping up to 0.5 V for each cycle.

For fabricating the Li‖LFP cell, the LiFePO_4_ cathode was prepared by mixing commercial LiFePO_4_ powder (80 wt%), carbon black (10 wt%) and PVDF (10 wt%) in an NMP solvent to form a slurry. The slurry was coated on an aluminum foil and then dried in a vacuum oven at 80 °C for 12 h. The electrolyte employed was 1.0 M LiPF_6_ in a mixture of EC (ethylene carbonate), DMC (dimethyl carbonate), and EMC (ethyl methyl carbonate) (volume ratio of 1 : 1 : 1). The cells were monitored in the galvanostatic mode within a voltage range of 2.0 and 4.0 V. The galvanostatic cycling test was conducted at 2.0C.

### 2.4 Characterization of materials

The morphologies of the samples were observed *via* field-emission scanning electron microscopy (SEM, Phenom Desktop SEM operated at 10 kV). The Brunauer Emmett Teller (BET)/Barrett Joyner Halenda (BJH) methods were employed for the determination of specific surface area and pore size distribution using an automated gas adsorption analyzer (Quantachrome). The functional groups in FSNSs were explored *via* Fourier transform infrared spectrometry (FTIR, PerkinElmer).

## 3. Results and discussion

**Fig. 1:**
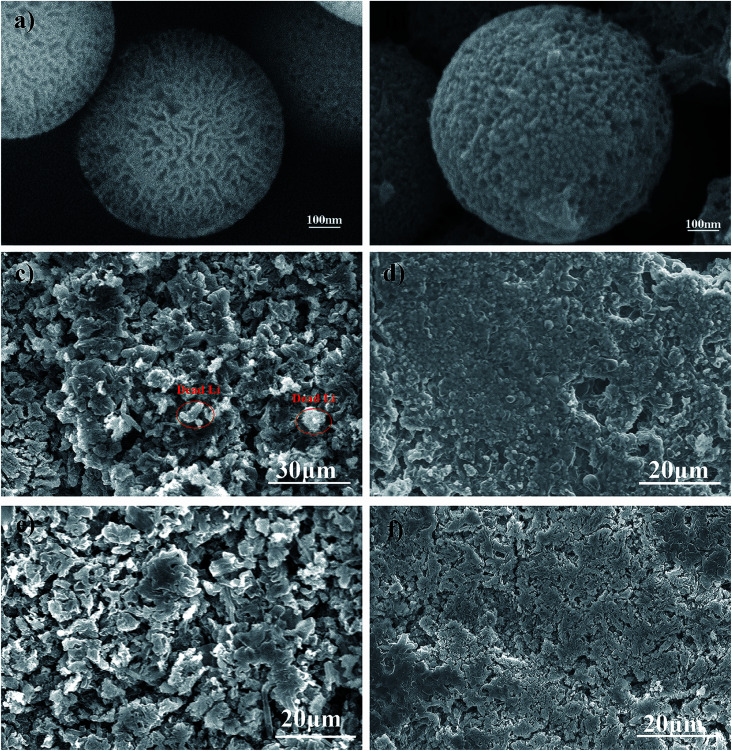
SEM images of Li deposition on the FSNSs and electrode surfaces at a current density of 0.5 mA cm^−2^ with different lithiation capacities. Li deposits on the FSNSs before (a) and after (b) 200 cycles, bare Li-metal electrode (c) and FSNS/Li composite electrode (d) with a total capacity of 0.5 mA h cm^−2^ after 200 cycles, bare Li-metal electrode (e) and FSNS/Li composite electrode (f) with a total capacity of 1.0 mA h cm^−2^ after 200 cycles.

FSNSs were fabricated *via* a direct and facile hydrothermal reaction. From Fig. S1,† it can be seen that the obtained silica nanospheres have rough surfaces and are about 600 nm in diameter. The zoomed-in image (Fig. 1a) reveals that there are many irregular pores on the surface of the nanospheres, and the pore size varies from several to tens of nanometers. The thickness of the FSNS coating was about 5.0 μm, as revealed by the cross-sectional SEM image (Fig. S2†). The N_2_ adsorption/desorption isotherm of FSNSs exhibited a typical IV isotherm with an H3 hysteresis loop (Fig. S3(a)†), suggesting the presence of mesopores. The specific surface area (BET) of FSNSs was 351.8 m^2^ g^−1^. The pore size (BJH) distribution of FSNSs is illustrated in Fig. S3(b)†, indicating a broad pore size distribution of mainly about 5 nm. This result is consistent with that observed in Fig. 1a. In the FTIR (Fourier transform infrared spectroscopy) spectrum (Fig. S4†), the absorbance peaks at around 1631 (470), 1098, 974 and 803 cm^−1^ correspond to the Si–OH bending vibration, Si–O–Si asymmetric stretching vibration, Si–O symmetric stretching vibration and O–Si–O asymmetric stretching vibration.

To verify the advantages of the porous silica nanosphere-coated separator in a long cycle test, the SEM images of the FSNS/Li composite anode before and after cycling were analyzed and compared with that of the bare Li-metal electrode. The Li‖Cu cells were cycled at a fixed current density of 0.5 mA cm^−2^ with a stripping/plating capacity of 0.5 mA h cm^−2^ for each cycle. After 200 cycles, the cells were disassembled in an argon (Ar)-protected glove box. Before SEM analysis, the electrodes were washed with DEC and dried completely. For one FSNS particle, it was observed that the FSNS was wrapped by lithium (Fig. 1a and b). This can be attributed to the adsorption of free lithium ions due to the strong interaction between the Li and O atoms on the surface of FSNSs and enough space for Li deposition within the porous structure during cycling.

At a current density of 0.5 mA cm^−2^ with a total capacity of 0.5 mA h cm^−2^, the surface of the bare Li-metal electrode became rough and porous as a result of the uneven deposition of lithium ions (Fig. 1c). Some dendrites and dead lithium (circled in red) were found on the surface of the bare Li-metal electrode (Fig. 1c). In contrast, under the same conditions, the FSNS coating impeded Li ion deposition around the high-current-density position, resulting in a relatively homogeneous lithium ion concentration and expediting the uniform deposition of lithium ions. Furthermore, it can be clearly seen in Fig. 1d that the surface of the FSNS/Li composite electrode is flatter without serious cracking and obvious dendrites. When the capacity was increased to 1.0 mA h cm^−2^ under the same current density, the composite electrode surface began to crack and a few mossy structures could be observed. Nevertheless, it was still superior to the bare Li-metal electrode (Fig. 1e and f).

**Fig. 2:**
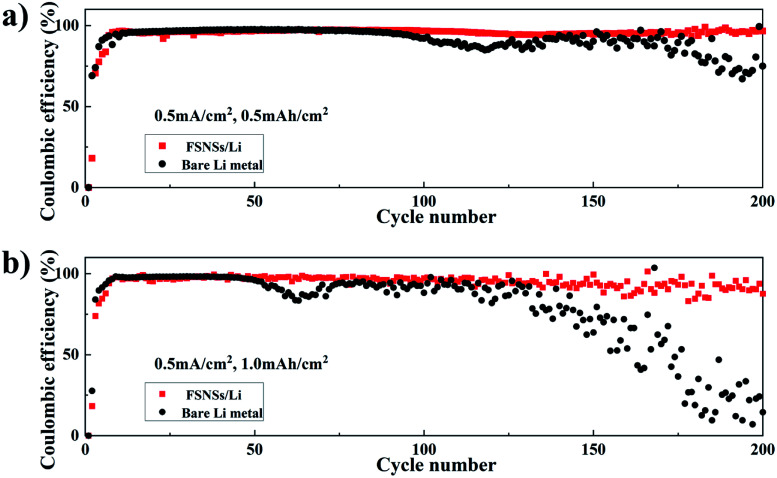
Comparison of the coulombic efficiency of Li deposition on the bare Li-metal electrode and FSNS/Li composite electrode at a current density of 0.5 mA cm^−2^ with a total capacity of (a) 0.5 mA h cm^−2^ and (b) 1.0 mA h cm^−2^.

To test the Li coulombic efficiency (CE), which is calculated based on the ratio of the amount of stripped Li to plated Li in each cycle and serves as a critical index to predict the cycle life of lithium-metal batteries (LMBs), Li‖Cu coin cells were fabricated using pieces of Cu foils as the working electrodes for Li metal plating. Fig. 2a and b compare the Li CE of the two Li‖Cu coin cells with and without the FSNS/Li composite electrode. The two cells were cycled with the total capacities of 0.5 and 1.0 mA h cm^−2^. At a lower capacity, *e.g.*, 0.5 mA h cm^−2^ (Fig. 2a), the CEs of the two cells were around 97% and comparable for the first 100 cycles. Subsequently, a fluctuation in the CE for the bare Li metal electrode became discernible, indicating the dendrite growth of Li and the formation of dead Li. When the FSNS/Li composite electrode was used, the CE slightly decreased to 95.9% after 200 cycles and the fluctuation was postponed to 170 cycles. Furthermore, upon increasing the capacity to 1.0 mA h cm^−2^ (Fig. 2b), the cell with the FSNS/Li composite electrode could still maintain higher CE, while the CE of the cell with the bare Li-metal electrode showed rapid decline after 52 cycles. For both cases, it is expected that the fluctuations in CEs should be more severe at higher capacity as a result of the accumulation of dead Li (Fig. 2). Nevertheless, the Li‖Cu cell with the presence of the composite showed improved stability upon electrochemical cycling and a longer service life. This improvement can be again ascribed to the pores and functional groups within FSNSs, which suppress the formation Li dendrites and dead Li. Additionally, the porous structure helps alleviate the volume change during the lithium-ion deposition/dissolution process.

**Fig. 3:**
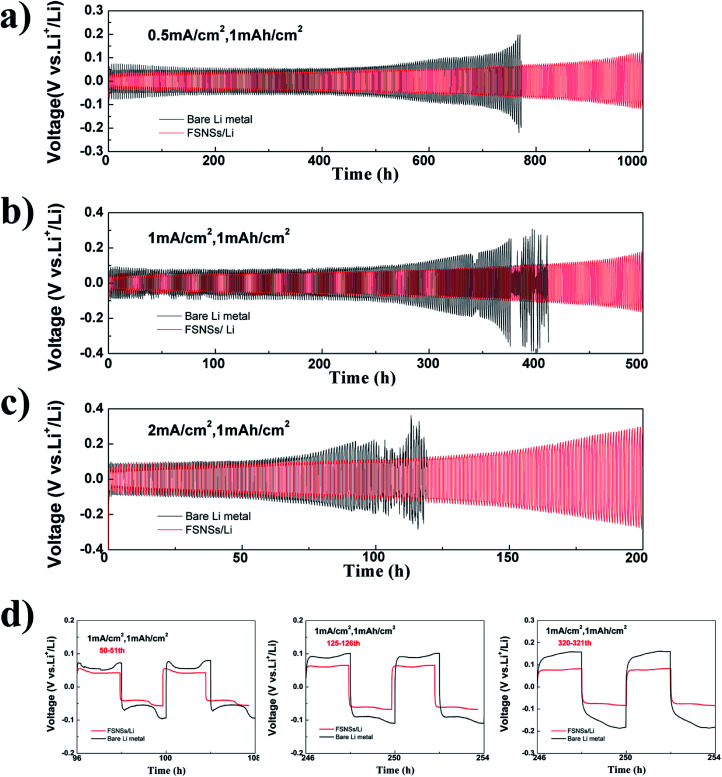
Galvanostatic cycling performance of the symmetric cells using the bare Li-metal electrode and FSNS/Li composite electrode at 0.5 mA cm^−2^ (a), 1.0 mA cm^−2^ (b), and 2.0 mA cm^−2^ (c) with a total capacity of 1 mA h cm^−2^; magnified Li plating/stripping profiles in the 50th, 125th, and 180th cycle under 1 mA cm^−2^ (d).

Li‖Li symmetrical cells were assembled for the galvanostatic cycling test. The cells were cycled with a fixed capacity of 1.0 mA h cm^−2^ at the current densities of 0.5, 1.0, and 2.0 mA cm^−2^. For the cell using the FSNS/Li composite electrode (Fig. 3a–c), its hysteresis (overpotential between Li deposition and dissolution) increased gradually with the increase in the number of cycles. Under a current density of 0.5 mA cm^−2^ (Fig. 3a), the symmetric cell delivered a stable voltage curve with a small hysteresis of about 55 mV within 600 h cycling. After 1000 h cycling, the hysteresis was only nearly 3 times the initial hysteresis, which was close to previously reported results.^31,45,46^ When there were no FSNSs, the cell showed a similar overpotential to that of the cell using FSNSs for the first 107 cycles; then, the hysteresis increased significantly after 121 cycles. After 191 cycles, it reached nearly 200 mV, which was higher than that of the cell using FSNSs. This increase in overpotential originated from the increase in the overall resistance as a result of the formation of dead Li.^47^ Similar scenarios (Fig. 3b and c) could be observed for the two cells running at higher current densities, *e.g.*, 1.0 mA cm^−2^ and 2.0 mA cm^−2^. It should be noted that sudden drops in the hysteresis occurred after 184 (379 h) and 102 (102 h) cycles for cycling at 1.0 and 2.0 mA cm^−2^, respectively. These phenomena can be attributed to the cell short-circuits caused by piercing the separator^31^. The advantage of using FSNSs was further supported by the detailed voltage profiles of the cycling plateaus at different cycles. The galvanostatic voltage profiles under different cycles for the two electrodes are compared in Fig. 3d. After the 50th cycle (100 h), the overpotentials were about 70 and 46 mV for the cells assembled without and with FSNSs, respectively. The corresponding profiles became 36 mV (100 mV *vs.* 64 mV) and 158 mV (250 mV *vs.* 92 mV) after 125 and 180 cycles.

**Fig. 4:**
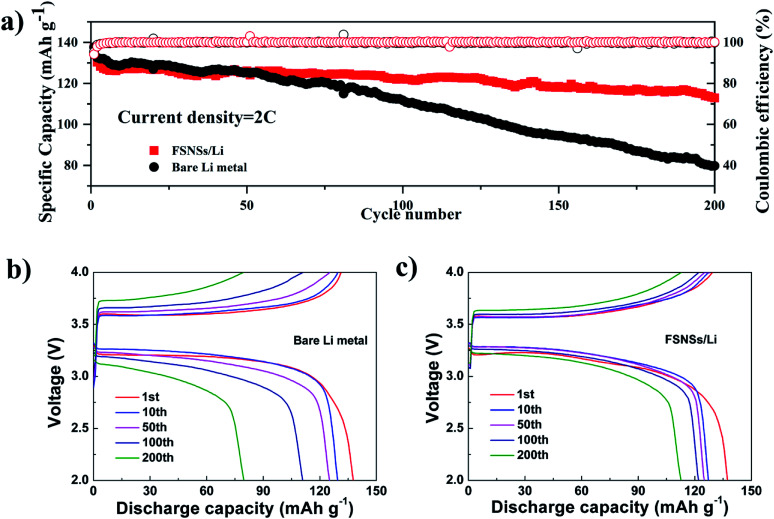
Cycling performance of the Li‖LFP cells using the bare Li-metal electrode and FSNS/Li composite electrode. (a) Long-term cycling performance of the Li‖LFP cells at 2C. Charge/discharge profiles after different cycles using (b) bare Li-metal electrode and (c) FSNS/Li composite electrode.

To further assess the application of the FSNS-modified electrode in real lithium-metal batteries, electrodes with and without FSNSs were assembled with an LFP cathode. The long cycling performance of the two cells was investigated under a rate of 2C (Fig. 4a). It was found that the capacity attenuation of the composite electrode was obviously better than that of the unmodified electrode. Fig. 4(b and c) show the specific capacity changes of the two electrodes at the 1st, 50th, 100th and 200th cycle. The first cycle discharge capacities of the two cells were similar and the values were 137.3 mA h g^−1^ and 137.7 mA h g^−1^ for the cells with the modified and unmodified Li electrodes, respectively. The capacity decline for the cell without FSNSs was attributed to the deposition of unstable Li ions and increased battery polarization, leading to a low capacity of 79.7 mA h g^−1^ (about 58% capacity retention) after 200 cycles. In contrast, the cell with the modified electrode delivered higher specific capacity (122.1 mA h g^−1^) and capacity retention (89%) for the initial 100 cycles, which degraded gradually to 112.7 mA h g^−1^ (82% capacity retention) after 200 cycles.

## 4. Conclusions

In summary, we reported a simple method for improving the cycling stability *via* forming FSNS/Li composite electrodes. The porous structure of FSNSs provided enough place for free lithium ion redeposition to inhibit the growth of lithium dendrites and the accumulation of dead lithium. As a result, the overpotential of the symmetrical battery with the FSNS/Li composite electrode could be improved in comparison to that of the bare Li-metal electrode at different current densities. The FSNS/Li composite electrode exhibited a high coulombic efficiency of 97% for the first 96 cycles, which was still maintained at 95% after 200 cycles at 0.5 mA cm^−2^. The galvanostatically cycling test on the Li‖LFP cells showed that the FSNS/Li composite electrode has excellent stability in the charging and discharging cycles. This FSNS/Li composite electrode provides a new method for limiting the growth of lithium dendrites and the formation of dead lithium.

## Conflicts of interest

There are no conflicts to declare.

## Supplementary Material

RA-010-C9RA09481D-s001
