# Supplementary material for: Protection of lithium anodes by fibrous silica nanospheres
Source: RSC Adv. 2020 Jan 20;10(6):3145–52. doi: 10.1039/c9ra09481d (PMC9048627; doi:10.1039/c9ra09481d)
Supplement: RA-010-C9RA09481D-s001 [file RA-010-C9RA09481D-s001.pdf]

Electronic Supplementary Material (ESI) for RSC Advances.
This journal is © The Royal Society of Chemistry 2019

Protection of Lithium Anode by Fibrous Silica Nanospheres

Jinxin Fan[a], Yu Luo[b], Keliang Jiang[a], Cheng Wang[a]*

[a] *Institute for New Energy Materials & Low-Carbon Technologies, School of Material Science and Engineering, Tianjin University of Technology, Tianjin, 300384, China*

[b] *Weifang Institute for Product Quality Inspection, Weifang, Shandong, 261000, China*

*Corresponding author

E-mail address: cwang@tjut.edu.cn

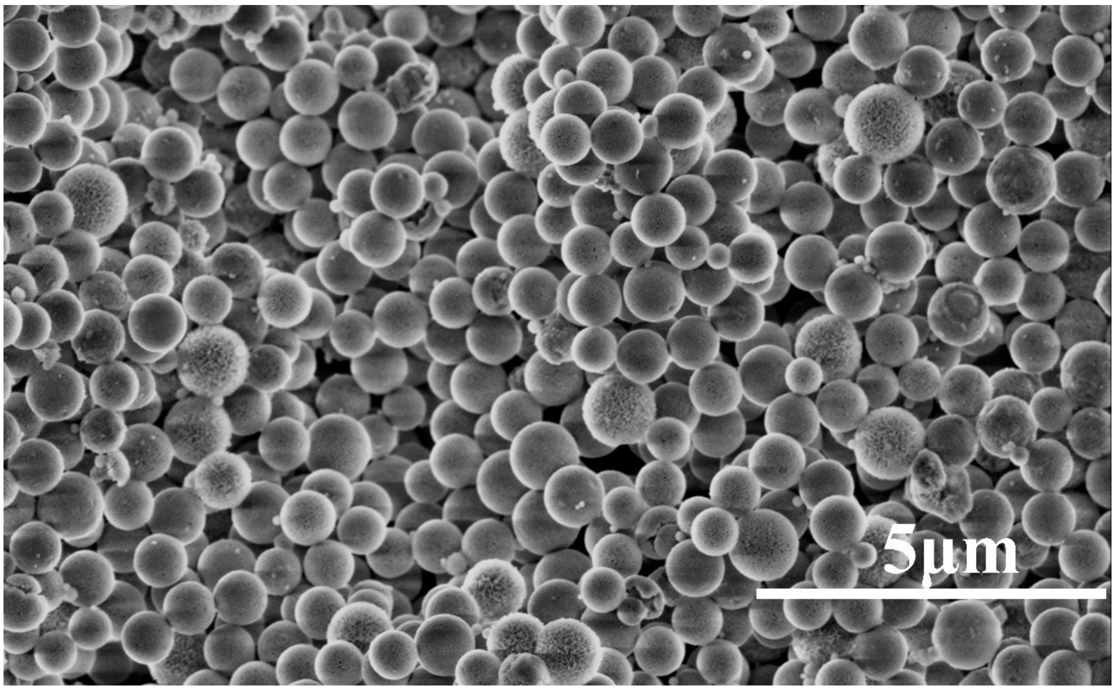

Figure S1. SEM image of FSNSs.

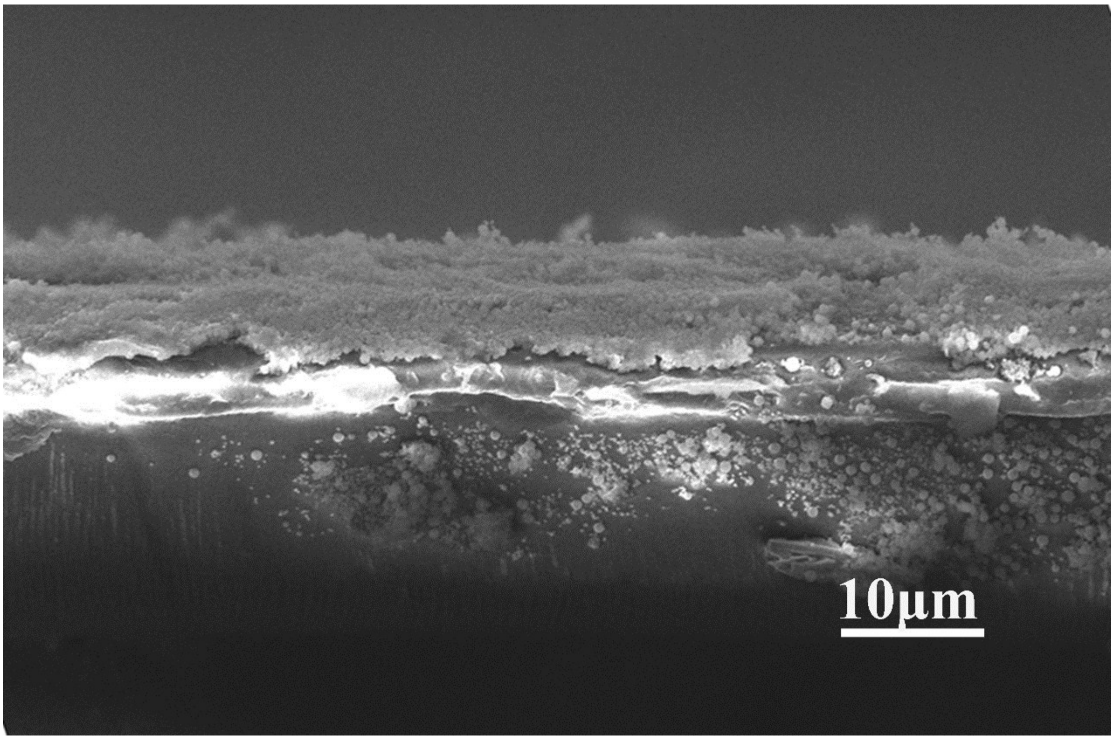

Figure S2. Cross-sectional SEM image of FSNSs coating.

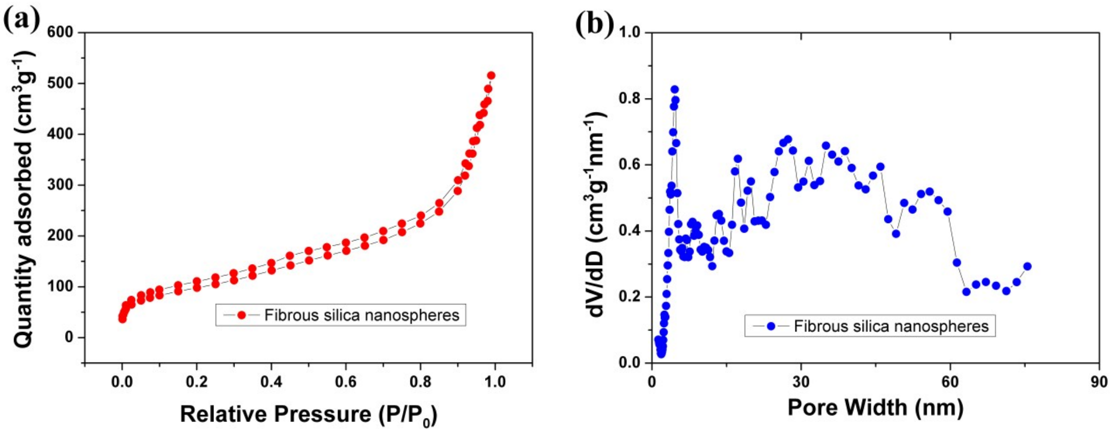

Figure S3 N_2 adsorption isotherms and pore size distribution of FSNSs.

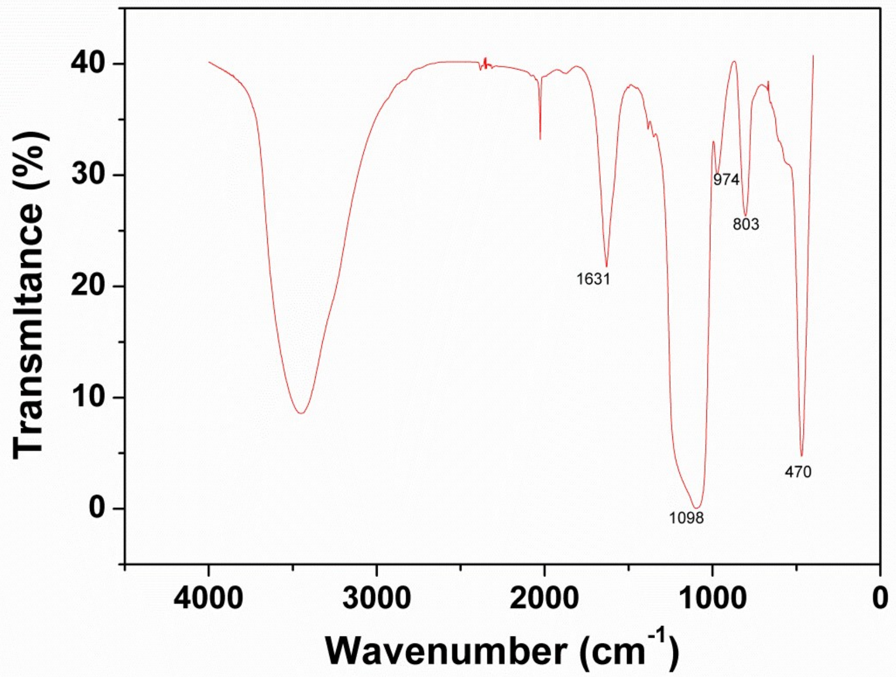

Figure S4 FTIR spectrum of FSNSs
